# Implementation and clinical effectiveness of a community-based non-communicable disease treatment programme in rural Mexico: a difference-in-differences analysis

**DOI:** 10.1093/heapol/czy041

**Published:** 2018-04-25

**Authors:** Kevin Duan, Ryan McBain, Hugo Flores, Francisco Rodriguez Garza, Gustavo Nigenda, Lindsay Palazuelos, Daniel Palazuelos, Elena Moreno Lázaro, Natán Enríquez Ríos, Patrick F Elliott

**Affiliations:** 1 Compañeros En Salud/Partners in Health Mexico, Calle Primera Poniente Sur No. 25, Ángel Albino Corzo, Chiapas, Mexico; 2 Department of Medicine, University of California San Francisco, San Francisco, CA, USA; 3 Partners in Health, Boston, MA, USA; 4 Division of Global Health Equity, Brigham and Women’s Hospital, Boston, MA, USA; 5 Harvard Medical School, Boston, MA, USA; 6 Instituto de Salud del Estado de Chiapas, Tuxtla Gutiérrez, Chiapas, Mexico

**Keywords:** Cardiovascular diseases, community health, diabetes mellitus, health systems, non-communicable disease, primary health care, Mexico

## Abstract

Non-communicable diseases (NCDs) account for the five largest contributors to burden of disease in Mexico, with diabetes representing the greatest contributor. However, evidence supporting chronic disease programmes in Mexico is limited, especially in rural communities. Compañeros En Salud (CES) partnered with the Secretariat of Health of Chiapas, Mexico to implement a novel community-based NCD treatment programme. We describe the implementation of this programme and conducted a population-based, retrospective analysis, using a difference-in-differences regression approach to estimate the impact of the programme. Specifically, we examined changes in diabetes and hypertension control rates between 2014 and 2016, comparing CES intervention clinics (*n* = 9) to care-as-usual at non-CES clinics (*n* = 806), adjusting for differences in facility-level characteristics. In 2014, the percent of diabetes patients with this condition under control was 36.9% at non-CES facilities, compared with 41.3% at CES facilities (*P* > 0.05). For hypertension patients, these figures were 45.2% at non-CES facilities compared with 56.2% at CES facilities (*P* = 0.02). From 2014 to 2016, the percent of patients with diabetes under control declined by 9.2% at non-CES facilities, while improving by 11.3% at non-CES facilities where the Compañeros En Salud Programa de Enfermedades Crónicas intervention was implemented (*P* < 0.001). Among hypertension patients, those with the condition under control increased by 21.5% at non-CES facilities between 2014 and 2016, compared with 16.2% at CES facilities (*P* > 0.05). Introduction of the CES model of NCD care was associated with significantly greater improvements in diabetes management between 2014 and 2016, compared with care-as-usual. Hypertension control measures were already greater at CES facilities in 2014, a difference that was maintained through 2016. These findings highlight the successful implementation of a framework for providing NCD care in rural Mexico, where a rapidly increasing NCD disease burden exists.

**Key Messages**

- Non-communicable diseases (NCDs) account for the five largest contributors to burden of disease in Mexico. Diabetes represents the greatest contributor to burden of disease, and this is only expected to worsen.
- Compañeros En Salud, a non-governmental organization, developed a four-pronged approach to NCD management [*Compañeros En Salud Programa de Enfermedades Crónicas* (CESPEC)] in rural Chiapas, Mexico.
- Based on a retrospective difference-in-differences analysis, CESPEC was associated with a significantly greater improvement in diabetic patients under control compared with care as usual at non-CES clinics throughout Chiapas. For hypertension patients, control rates were already greater at CES clinics at baseline compared with non-CES clinics, a difference that was maintained through 2016.

## Introduction

Mexico has experienced a significant change in disease burden over the last 30 years and now faces a looming health crisis in the form of chronic, non-communicable diseases (NCDs) (Gómez-Dantés *et al*. 2016). Although great strides have been made to reduce morbidity and mortality related to malnutrition and communicable diseases, the epidemiologic shift in burden of disease towards NCDs is a major cause for concern (Stevens *et al*. 2008). Based on a recent study, NCDs now account for all of the top five highest contributors to burden of disease in Mexico, with diabetes as the single greatest cause of disability adjusted life years in the country (Institute for Health Metrics and Evaluation n.d.; Kassebaum *et al*. 2016). This is only projected to worsen with time (González-Pier *et al.* 2016).

Efforts are in place throughout Mexico to tackle the rising NCD burden. The Mexican government implemented the *Seguro Popular* health insurance system in 2003 that allocated an unprecedented level of new funding to states with the promise of universal health care. Along with this effort, the ‘Units Specialized in the Treatment of Chronic Diseases’ programme was introduced as a chronic disease management referral programme in urban areas aimed to improve outcomes in NCDs (Córdova-Villalobos *et al.* 2008; Knaul *et al.* 2012). However, these interventions have poor reach into rural areas, where access to health care is limited at best, and can often be nonexistent (Salinas *et al.* 2010). This is despite the fact that the NCD disease burden in rural areas of Mexico is similar to that of urban areas (Hernández Ávila *et al.* 2016; Oyebode *et al.* 2015).

Compañeros En Salud (CES), the Mexico-based affiliate of Partners In Health, is a non-governmental organization that aims to improve access to services and build an approach of high-value, comprehensive primary healthcare in rural Mexico. Since 2011, CES has been developing a novel approach of NCD care—known as the *Compañeros En Salud Programa de Enfermedades Crónicas* (CESPEC)—in collaboration with the Secretariat of Health of Chiapas (SSCH in Spanish). Based on prior experiences in the region (Molina and Palazuelos 2014) and existing best practices (Bhutta *et al.* 2010; Dolea 2010), the approach encompasses a new cadre of community health workers, supply chain improvements, active case-finding, and a unique education-support model for rural providers. To evaluate this approach, we measured change in patient outcomes among hypertension and diabetes patients before and after implementation of the programme, compared with regional-level trends in outcomes throughout all of Chiapas. We hypothesized that CESPEC would result in significantly greater improvement in diabetes and hypertension control among those enrolled in care at CES facilities over the study period, compared with rural non-CES facilities throughout Chiapas.

## Materials and methods

### Setting

Since 2011, CES has worked in the Sierra Madre region of Chiapas, one of the poorest states in Mexico, with 76% of the population living in poverty (OECD, 2015). Historically, Chiapas has performed poorly on public health metrics and has had one of the lowest life expectancies and highest maternal mortality rates of any state in the country (OECD, 2015; Gómez-Dantés *et al.* 2016). CES collaborates with the SSCH and within the existing health infrastructure to operate eleven rural clinics in the region across two health jurisdictions. These clinics cover a population of about 25 000 individuals. Health care is free to patients at the point of delivery, since these populations fall in the lowest income deciles defined by *Seguro Popular*.

The status quo of health care in rural Mexican communities is heterogeneous: Although most communities have small health stations or clinics, there are varying levels of staffing, medical training and supplies. As a result, many communities have no functioning clinic and the population must travel long distances to consult an appropriately trained medical provider (Secretaría de Salud 2009). Those communities that do have a functioning healthcare facility with an appropriately trained medical provider usually experience episodic care, even for chronic diseases. Although the SSCH attempts to provide longitudinal care, the reality is that there is a lack of continuity due to distrust in providers and care fragmentation (Molina and Palazuelos 2014). In general, patients seek health care for acute illnesses or for chronic diseases on an as-needed basis (e.g. a lack of medications).

### Implementation

In contrast to the status quo of primary care delivery in rural Mexico, CES has implemented a novel four-pronged approach. First, provision of primary care is by *pasantes*. These are medical providers completing their social service year (*pasantia*), which is required before being able to obtain a medical degree. *Pasantes* often staff rural and marginalized communities, acting as the de facto safety net (Laveaga 2013). However, supervision is inadequate and the quality of care delivered is variable (Nigenda 2013). As a way to recruit *pasantes* to rural Chiapas and provide the educational and supervisory support that is currently lacking, CES developed a medical and global health curriculum, as well as a system of clinical supervision to fill the void (Van Wieren *et al.* 2014). This provides enhanced training on the medical management of NCDs.

Second, each CES community is supported by a cadre of community health works known as *acompañantes*. They are local community members nominated by the community and trained/mentored by CES staff to provide support to patients with chronic diseases, including adherence support through home visits and accompaniment to routine, monthly or bimonthly clinic visits with the clinic provider. This strengthens the longitudinal relationship of patients with the healthcare system. The *acompañantes* are reimbursed for their work with monthly food packages provided by CES.

Third, CES developed a logistical support and supply chain management system to minimize the frequent medication and supply stock-outs previously experienced. This includes inventory management and distribution solutions to maintain full availability of the most highly utilized medicines. Clinic providers identify inventory requirements each month, and CES staff fulfils these requirements through either the existing medication supply chain provided by the SSCH or private purchase. All medications purchased privately are medications approved for use at primary level clinics on the ministry’s formulary, but frequently stock-out.

Finally, active case-finding campaigns led by CES supervisors, *pasantes* and volunteers take place to identify cases of diabetes and hypertension (among other conditions) and integrate these new patients into care. These campaigns are conducted biannually with four out of ten communities screened each year. During the case-finding campaigns, providers go door-to-door over a week-long period and conduct a pre-determined set of health screenings. This is done in conjunction with local community involvement and knowledge, such that every household in the community is screened.

**Figure 1.**
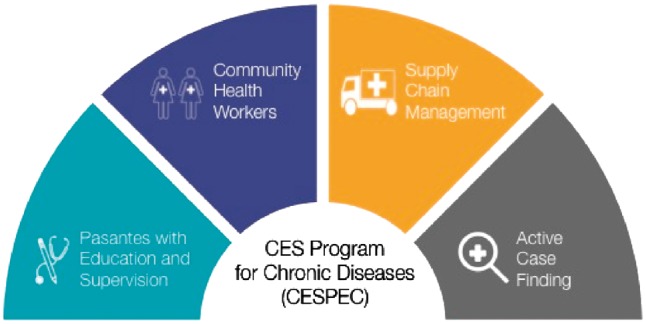
CESPEC approach of NCD care

This four-pronged approach was piloted then rolled out, in 2014. Figure 1 provides an overview of this model.

Monitoring and evaluation of the implementation process was conducted monthly to assess the fidelity, reach and dose of the intervention (Moore *et al.* 2015). Process metrics collected to assess the dose of the CESPEC implementation included the screening data from the active case-finding programme, as well as CHW uptake figures. Process metrics collected to assess the fidelity of the CESPEC implementation included measuring clinic visit attendance and missing disease control data at follow-up visits.

The implementation of CESPEC required additional investments beyond usual care, including hiring clinical supervisors to teach and train *pasantes*, staff to develop and run the CHW programme and the private purchase of medications to supplement the SSCH supply. A detailed analysis of the resources and costs required to implement CESPEC is beyond the scope of this study, and will be addressed in a future cost-effectiveness evaluation. A preliminary estimate is that CES invests around USD$150 per patient per year to run the CESPEC programme.

### Study design

We performed a retrospective, population-based analysis throughout the entirety of the Chiapas region of Mexico, from 2014 to 2016. All components of CESPEC described above were implemented in nine of eleven clinics by 2015. As such, these nine clinics (*n* = 9) were included as the study population, with each clinic representing the unit of analysis. Clinical outcomes were collected from the CES electronic health records (EHRs) for a pre-intervention baseline period of 8 months from January to August 2014, before implementation of the model. To assess the intervention effect, post-intervention data were collected 2 years later (2016) during the same months. In total 2 of the 11 clinics which CES operates were opened in 2015, and were therefore excluded from this study due to lack of availability of pre-intervention baseline data. Due to the remoteness of each clinic (average driving distance to the nearest non-CES clinic is 2.5–3 h by poorly maintained dirt roads requiring four-wheel drive vehicle), spillover of the intervention between communities was assumed to be minimal.

The comparison group was all other SSCH clinics in Chiapas over the same study period, representing 806 facilities (*n* = 806)—providing coverage for over three million individuals. These data were obtained from a SSCH health database which is publicly accessible online (Secretaría de Salud n.d.). Clinics located in the capital city of each state health jurisdiction were removed from this group to exclude urban clinics and better match the rural patient population of CES clinics. Thus, overall inclusion criteria comprised: (1) SSCH clinics in Chiapas outside of jurisdiction capitals, which were functional between 2014 and 2016, (2) adults 18 years of age or older and (3) patients with a confirmed diagnosis of diabetes or hypertension.

Sociodemographic data at the patient- or clinic-level were not available in the datasets used for the data analysis. Therefore, as a proxy, municipality-level census data were used from 2015 to compare the sociodemographic characteristics of CES municipalities and non-CES municipalities (Instituto Nacional de Estadística y Geografía n.d.).

The study was approved by the Partners Healthcare Institutional Review Board (2017P000400/PHS) as exempt due to the retrospective evaluation of existing data in a de-identified format, and also approved by the Bioethics Committee of the State of Chiapas.

### Outcome measures

We chose two primary outcomes: the percent of patients with diabetes in control, and the percent of patients with hypertension in control. The term ‘in control’ was operationalized based on SSCH definitions. For diabetes, SSCH considers control during a given month as a haemoglobin A1c below 7%, or (if not available) a fasting blood glucose level below 7.16 mmol/l (130 mg/dl). For hypertension, the SSCH considers control during a given month as a systolic blood pressure (SBP) below 140 mmHg and diastolic blood pressure (DBP) below 90 mmHg, and for diabetics, an SBP below 130 mmHg and a DBP below 80 mmHg.

The percent of individuals with disease under control was measured as the number of diabetes and hypertension patients at the facility-level who met these definitions in a given month, relative to the total number of patients seen for these conditions over the same time period. We compared 8-month averages, at the facility-level, in 2014 and 2016 in order to examine change over time.

### Data analysis

We examined statistical power to detect a medium effect size (δ = 0.50) when comparing facility-level trends among CES facilities vs facilities throughout all other municipalities in Chiapas region, over the 2-year period. Assuming a standard α-level of 0.05, sample of 800 facilities and serial correlation of *r* = 0.50, statistical power was greater than π = 0.80.

We conducted a difference-in-differences (DIDs) regression approach to account for baseline differences between the intervention and control groups. We used mixed-effects, multi-level models to evaluate NCD outcomes across facilities and to compare performance among CES facilities and all other facilities. Random effects were incorporated for facilities and municipalities, whereby years were nested within facilities, and facilities were nested within municipalities. Fixed effects were included for CES facility and for the year of analysis—in order to examine change in 2016, relative to the baseline year of 2014. The interaction term of interest was CES × year, which examined whether change over time on outcomes of interest was greater among CES facilities receiving the intervention package, vs all other facilities. Facility-level covariates included the percent of patients who were female, the percent of patients who were over 60 years of age, and the total number of visits at a facility in a given month—a proxy for facility size.

In addition to the overall analysis, sub-group analyses were performed in order to examine whether there were differential effects among men vs women and younger (ages 20–59) vs older (ages 60 and over) individuals. To compare sociodemographic characteristics at the municipality-level, we conducted two-sample t-tests. All analyses were conducted in Stata version 13.0 SE (StataCorp 2013).

## Results

### Process evaluation

From 2014 to 2016, process metrics to evaluate the dose of the CESPEC implementation demonstrated increased involvement of CHWs with diabetes and hypertension patients, as well as increased screening of individuals in the active case-finding programme. Metrics designed to assess the fidelity of the CESPEC implementation such as percentage of patients with missing disease control information in the EHR showed overall improvement from 2014 to 2016. Table 1 contains a full description of process evaluation data collected during the implementation of CESPEC.

**Table 1.** Overview of process evaluation metrics collected during CESPEC implementation—2014–16

Process metric	2014	2015	2016
Dose			
Active case-finding			
Individuals screened	936^a^	2234	3095
Referrals to clinic due to positive screens	410^a^	575	1017
Diabetes patients followed by CHW	76	84	105
Hypertension patients followed by CHW	100	103	123
Fidelity			
Missing diabetes control data in EHR	16.5%	11.5%	10.7%
Missing hypertension control data in EHR	8.2%	3.8%	6.0%
Attendance at diabetes follow-up appointment	82.4%	61.4%	75.0%
Attendance at hypertension follow-up appointment	76.7%	57.7%	73.7%

a Figures for 2014 are missing data from one of the two case-finding campaigns during that calendar year.

### Descriptive statistics

In 2015, the municipalities in which CES communities are located demonstrated a similar level of socioeconomic status to that of non-CES municipalities. The only socioeconomic indicator that had a statistically significant difference was car access, with 19.5% of households in CES municipalities having access to a vehicle, compared with 11.3% in non-CES municipalities (*P* = 0.047). A full description and comparison of sociodemographic characteristics of CES municipalities and non-CES municipalities can be found in Table 2.

**Table 2.** Comparison of sociodemographic characteristics between CES municipalities vs non-CES municipalities—2015

Variable	CES (*n* = 3)	Non-CES (*n* = 105)	*P*-value
Education (% of population)			
Adult literacy rate	81.5%	80.1%	0.75
Highest level of education			
No formal education	15.7%	18.1%	0.56
Primary school	61.9%	61.8%	0.97
Secondary school	37.3%	37.7%	0.93
Higher education	3.0%	5.0%	0.37
Employment (% of population)			
Employed with a salary	34.3%	44.3%	0.43
Agricultural worker	66.3%	56.0%	0.41
Income below minimum wage	61.7%	46.9%	0.20
Economically active	34.1%	39.4%	0.11
Housing (% of households)			
Dirt floor in house	14.1%	13.6%	0.92
Wood/coal as cooking fuel	84.7%	72.3%	0.30
Gas as cooking fuel	13.6%	25.7%	0.30
Access to car	19.5%	11.3%	0.047
Access to telecommunications			
Internet	1.9%	3.5%	0.48
Computer	3.7%	5.4%	0.47
Cellular telephone	38.0%	44.7%	0.59
Fixed line telephone	2.7%	4.9%	0.34
Television	73.1%	65.4%	0.50

In total, the monthly average for patients enrolled in care at SSCH clinics from January to August 2014 was 14 272 hypertension patients and 13 010 diabetes patients across 806 facilities. This declined to 8089 hypertension patients and 8250 diabetes patients across 806 facilities in 2016. This compared with an increase in patient volume at CES clinics over the same period.

At baseline, the percentage of patients that were women in 2014 was higher at CES clinics compared with non-CES clinics. This difference was maintained in 2016. There was no observed difference in the percentage of patients over age 60 that attended CES vs non-CES clinics in either 2014 or 2016.

In terms of management of these conditions, 45.2% of hypertensive patients had blood pressure under control in 2014 at non-CES clinics, compared with 66.7% in 2016. Among diabetes patients at non-CES clinics, those with appropriately managed glucose levels were 36.9% in 2014 and 27.7% in 2016. A fuller overview of descriptive statistics, comparing CES and non-CES facilities between 2014 and 2016, can be found in Table 3.

**Table 3.** Overview of hypertension and diabetes population in Chiapas—2014 vs 2016

Population	2014			2016		
Hypertension	CES (*n* = 92)	Non-CES (*n* = 14 272)	*P*-value	CES (*n* = 196)	Non-CES (*n* = 8089)	*P*-value
Female	59.8%	74.0%	0.002	60.1%	77.2%	<0.001
Age 60+	52.3%	48.7%	0.50	49.7%	52.0%	0.49
Under control	56.2%	45.2%	0.03	72.4%	66.7%	0.09
Diabetes	CES (*n* = 43)	Non-CES (*n* = 13 010)	*P*-value	CES (*n* = 134)	Non-CES (*n* = 8250)	*P*-value
Female	64.5%	72.0%	0.32	68.3%	77.9%	0.006
Age 60+	22.0%	32.7%	0.10	32.0%	34.5%	0.55
Under control	41.3%	36.9%	0.50	52.6%	27.7%	<0.001

CES stands for *Compañeros En Salud* facilities. Categorical variables presented as monthly average enrolment size for the given year, and percent of total enrolments according to population characteristic. Figures presented here are descriptive changes, unadjusted for facility-level differences between CES and non-CES facilities.

### Mixed-effects multilevel models

For mixed-effects models examining hypertensive patients, the average non-CES facility demonstrated successful blood pressure management in 37.3% of cases that presented over the course of 2014. We found a main effect for facility type (β = 0.203, *P* = 0.022, 95%CI: 0.030, 0.377), indicating that there was higher baseline management of hypertension in CES facilities compared with non-CES facilities. There was also a significant main effect for the percentage of individuals over 60-years old (β = 0.052, *P* = 0.004, 95%CI: 0.017, 0.088), connoting that a 1% increase in the percentage of the population at a facility over 60-years old was associated with a 5.2% increase in successful blood pressure management. Main effects for percentage of the population that was female, and total number of facility visits, were non-significant (*P* > 0.05).

The overall effect of time was significant (β = 0.230, *P* < 0.001, 95%CI: 0.210, 0.249), demonstrating a secular trend of improved blood pressure management between 2014 and 2016. The magnitude of this change, when comparing CES to non-CES facilities was smaller among CES facilities, but this result was not statistically significant (β = −0.088, *P* > 0.05, 95%CI: −0.177, 0.001). In other words, hypertension control rates increased by 14.2% in CES facilities from 2014 to 2016 and also increased by 23.0% in non-CES facilities.

For mixed effects models examining diabetes patients, the average non-CES facility demonstrated successful blood-glucose level management in 26.9% of cases that presented over the course of 2014. Main effects were observed for age (β = 0.044, *P* = 0.009, 95%CI: 0.011, 0.076) and sex (β = 0.106, *P* < 0.001, 95%CI 0.068, 0.143), indicating better outcomes at facilities with greater proportions of women and older adults. Adjusting for demographic variables, there was no significant difference between CES and non-CES facilities in 2014 (*P* > 0.05).

The overall effect of time was significant (β= −0.076, *P* < 0.001, 95%CI: −0.094, −0.058), demonstrating a trend of reduced levels of blood glucose management between 2014 and 2016. This trend significantly differed at CES facilities, which observed improvement in management levels over this period (β = 0.204, *P* < 0.001, 95%CI: 0.121, 0.287). In other words, diabetes control rates increased by 12.8% in CES facilities from 2014 to 2016, while decreasing by 7.6% in non-CES facilities.

## Discussion

**Figure 2.**
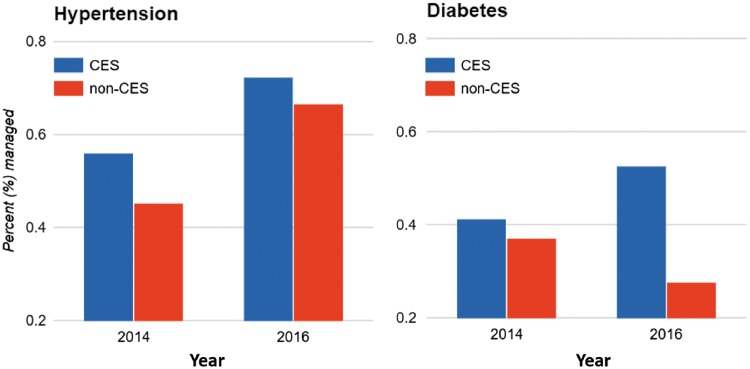
Diabetes and hypertension management, CES and non-CES facilities. CES stands for *Compañeros En Salud* facilities. Percent managed represents the average percentage of patients, across CES or non-CES facilities for a given year, whose condition has been stabilized and is under control, based on national guidelines. These figures, derived from statistical models, have been adjusted for facility-level differences in patient characteristics

We find that introduction of a novel model of NCD care (CESPEC) in rural Mexico significantly improved diabetes management, while care-as-usual in neighbouring facilities observed declines in diabetes management over the same time period. Specifically, diabetes control rates improved by 12.8% in CES facilities from 2014 to 2016, while declining by 7.6% in non-CES facilities, after adjusting for facility-level differences. In absolute terms, control rates among diabetes patients at CES facilities in 2016 were more than double the average at clinics throughout the rest of Chiapas. To our knowledge, this is the first study to demonstrate a significant effect of any programme in rural Mexico that improves diabetes outcomes (Figure 2).

Regarding hypertension management, we found that both CES and non-CES facilities improved between 2014 and 2016. Although this difference was not statistically significant, hypertension control rates at CES facilities were already 20 percentage points higher at baseline, a difference that was highly statistically significant. This higher baseline control for hypertension may represent better quality of care due to the presence of CES prior to the full implementation of CESPEC that specifically targeted NCD control, though we attempted to control for this using DIDs regression approach. Interestingly, this same effect was not noted in diabetes, suggesting that the prior presence of CES had a variable effect and there may be differences inherent in the two disease processes that accounts for this. An alternative explanation is that the ability for CES facilities to excel even further between 2014 and 2016 was undercut by the pre-existing higher baseline. Nevertheless, CES clinics out-performed non-CES clinics in terms of absolute clinical control, improving 16.2% and achieving 72.4% control in 2016. This compares to the Chiapas average of 66.7% in 2016, the national average in Mexico of 58.7%, and the US national average of 54% (Hernández Ávila *et al.* 2016; Yoon *et al.* 2015).

There are several likely reasons for the marked improvement in diabetes care under CESPEC. As concluded in previous studies and reflected in current guidelines, the CES model utilizes a multidisciplinary and multifactorial approach to disease management (Ricci-Cabello *et al.* 2013; International Diabetes Federation Guideline Development Group 2014). This includes organizing multiple providers (physicians and CHWs in CESPEC) to care for the patient and focussing on both medical and lifestyle modification as treatment modalities. In addition, the CES approach represents a shift from episodic care to longitudinal, community-based primary care, with both providers and CHWs following patients with chronic diseases on a monthly basis. Longitudinal care is rare and limited in the Sierra Madre region of Chiapas, and sometimes does not exist in other parts of Mexico, urban or rural. Best evidence demonstrates that continuity of care is essential for improving chronic disease outcomes, especially for diabetes (Mainous *et al.* 2004).

The active case-finding employed by CESPEC likely also played a role in the improved outcomes for both hypertension and diabetes. The effect of case-finding is evident in the gender breakdown for CES clinics compared with non-CES clinics, with CES clinics serving a statistically significant higher proportion of men. For a variety of reasons, healthcare seeking behaviour has been observed in multiple settings to be generally greater among women than men (Read and Smith 2017). Active case-finding may be helping bring more men with NCDs into care at CES, which could have major implications for population health in the region.

In terms of human resource development, the model leverages an existing network of general medicine providers (such as the *pasante* role in CESPEC) that already cares for rural patients in Mexico. Rural providers often suffer from a lack of supportive supervision and continued training, which limits the ability of these providers to deliver high-quality care (Gutiérrez and García-Saisó 2016). The CESPEC education-support curriculum addresses this issue, and also fosters a strong link between the provider and the community that is absent in standard care models (Segall 2000; Hsin and Macer 2004; Budd 2007). The improvements in supervision and support that CESPEC provides, and the associated outcomes demonstrated in this study, are evidence that high-quality rural care is achievable with general medicine providers in Mexico.

CHWs have been utilized in other Mexican care delivery models. Previously published examples of CHWs in Mexico generally focus on disease-specific prevention (Gaziano *et al.* 2015; McClellan and Tapia Conyer 2015; Balcazar *et al.* 2016). The CESPEC approach represents a shift in scope from these isolated preventative approaches to an integrated patient care approach that positions CHWs as core members of the care delivery team. These CHWs receive investments in their recruitment, training and reimbursement so that they will be retained long-term. CES is currently completing a stepped-wedge trial quantifying the clinical effect that can be attributed from CHWs incorporated in these ways (Newman *et al.* 2015).

The CES approach to rural primary care enhances clinical effectiveness by reorganizing existing inputs in the Mexican health system, rather than creating a parallel system. This points to the scalability of the CESPEC model, or at least certain components of the model, and suggests that the added costs and resources needed to implement CESPEC are targeted investments that could be layered into the broader health system over time. We plan to pursue subsequent analyses that will focus on the costs and cost-effectiveness of CESPEC, relative to care-as-usual. As demonstrated in other settings, greater up-front investments in chronic disease management are likely to prevent the costly sequelae of late-stage disease (World Health Organization and Public Health Agency of Canada 2005; Nussbaum 2006; Li *et al.* 2010).

There are several key limitations to this study. First, this was a retrospective study utilizing a quasi-experimental design. As such, there are biases that cannot be fully eliminated, short of a randomized study. Specifically, although we used a DIDs regression approach to account for baseline differences in performance characteristics, it is possible that higher baseline levels of infrastructure and improved baseline disease control led to catalytic improvements during the implementation of CESPEC that are not accounted for in our study design. This could have overestimated the effect of the intervention. Furthermore, matching of intervention and comparison groups was limited by the demographic characteristics available within the government database. Thus, our ability to account for selection bias was limited, and may have also led to an overestimation of the intervention effect size.

Second, the length of our study period was 2 years. Diabetes and hypertension are chronic conditions, and therefore it will be important to demonstrate if the observed outcomes can be sustained long-term. Third, this was a regional study conducted in one Mexican state. Thus, the generalizability of these findings should be interpreted with caution. Fourth, the data was obtained from two different databases, and as such, there are potential differences in data quality that introduces another possible source of selection bias that could be not be controlled for during the data analysis.

Last, there was a reduction in reported patient volume among non-CES clinics between 2014 and 2016, while CES clinics grew in terms of patient volume. One theory explaining the observed decrease in volume in non-CES clinics is that anecdotally, the paperwork burden in government clinics is extremely high, and as such providers do not always enter patient control data into the government database in a reliable or consistent manner. Furthermore, in rural areas, the information technology infrastructure is limited and it is not always possible to enter data into the system due to connectivity issues. This may have resulted in selection bias in the government data source if patient control data omission was non-random. However, one would expect that any such bias would skew the data towards improved non-CES clinic performance, and therefore, would likely underestimate the effect of the CESPEC intervention. As such, the overall impact of this observation on the analysis is unclear.

The CESPEC approach fills a void in the existing Mexican healthcare system by providing high quality and clinically effective care to one of the most marginalized populations in the country. By improving outcomes in diabetes and hypertension, this intervention offers a potential approach to NCD care that could be reproduced, piloted and adapted in other regions of Mexico as a way to combat the rapidly increasing scale of NCDs throughout the country.
